# Valproate-induced hyperammonemic encephalopathy: a case report

**DOI:** 10.1186/s13256-020-2343-x

**Published:** 2020-01-25

**Authors:** Suhal Shah, Richard Wang, Ulrick Vieux

**Affiliations:** Orange Regional Medical Center, Middletown, NY USA

**Keywords:** Valproate, Valproic acid, Hyperammonemia

## Abstract

**Background:**

Hyperammonemic encephalopathy is a rare and serious adverse reaction to valproate. Although there is documentation of this reaction in previous reports, very little is still known about the exact mechanism of action. In addition, there are no established guidelines of the next steps needed when a patient does develop this reaction. Therefore, this case report highlights what is known as well as the areas of research still needed.

**Case presentation:**

Our patient was a 57-year-old Caucasian woman with a medical history of bipolar I disorder, opioid use disorder, benzodiazepine use disorder, and Crohn’s disease who was admitted to our behavioral health unit for suicidal ideation. She had been experiencing multiple panic attacks for 2.5 weeks along with poor sleep, increased energy, excessive spending, and feelings of helplessness. The patient was diagnosed with bipolar I disorder, manic episode without psychotic features, and benzodiazepine use disorder. She was started on valproic acid, citalopram, propranolol, and quetiapine. By day 6 of her hospitalization, the patient had altered mental status, varying levels of consciousness, confusion, and ataxic gait. Her ammonia levels were found to be elevated. All of her medications were discontinued, and lactulose was initiated. She returned to her baseline mentation within 48 hours and was discharged with lithium and quetiapine. The treatment team concluded that this patient had valproate-induced hyperammonemic encephalopathy, a rare but reversible reaction to valproate.

**Conclusion:**

Fortunately, rapid identification of this rare condition led to a favorable outcome in our patient. This case report illustrates the course of treatment in a patient who experienced this reaction and reviews current knowledge as well as areas of needed research in regard to valproate-induced hyperammonemic encephalopathy.

## Background

Divalproex sodium/valproic acid (VPA) is used with therapeutic plasma blood levels between 45 and 125 μg/ml to treat complex partial seizures, simple and complex absence seizures, migraines, bipolar mania, and schizoaffective disorders. The most commonly known side effects include sedation; dizziness; dose-dependent tremors; ataxia; headaches; and gastrointestinal side effects, including weight gain, abdominal pain, nausea, vomiting, diarrhea, constipation, and reduced appetite [1]. Another rare adverse reaction is reported in multiple published cases highlighting VPA-induced hyperammonemic encephalopathy (VHE) in the setting of normal liver function tests, which, when identified immediately, is reversible.

## Case presentation

A 57-year-old Caucasian woman with a medical history of bipolar I disorder, previous psychiatric hospitalizations, opioid use disorder, benzodiazepine use disorder, and Crohn’s disease as well as a family history of bipolar I disorder presented to the psychiatric emergency department with worsening anxiety and depressive symptoms. After a recent relationship breakup, she had begun experiencing multiple panic attacks and suicidal ideation, prompting her sister to call 911. She was admitted to the behavioral health unit of our hospital on an involuntary basis. Her admission laboratory test results were positive for benzodiazepines by urine toxicology.

During her intake interview after admission, she reported decreased need for sleep, poor appetite, racing thoughts, increased energy level, panic attacks, anxiety, excessive spending, and feelings of helplessness. She denied any suicidal ideation, auditory/visual hallucinations, or paranoid thoughts. Her mental status examination revealed that she appeared to be her stated age, was casually dressed with good hygiene, and maintained good eye contact. She was cooperative during the interview process but provided tangential responses. Along with a constricted affect and anxious mood, she presented with rapid and pressured speech, increased distractibility, poor concentration, and lack of insight and judgment. She was diagnosed with bipolar I disorder, manic episode without psychotic features, and benzodiazepine use disorder. The treatment team began her on a trial of VPA 500 mg twice daily by mouth for her mood, citalopram 20 mg by mouth once daily for her anxiety, propranolol 20 mg twice daily by mouth for panic attacks, and quetiapine 200 mg by mouth once nightly for sleep. Her dose of propranolol was increased to 30 mg twice daily by mouth. Her VPA level drawn on day 4 was within therapeutic range, and she appeared less euphoric and less pressured. She denied experiencing any side effects from her medication, but on day 6, she was found to have a sudden onset of altered mental status, waxing and waning level of consciousness, confusion, lethargy, and ataxic gait. Upon physical examination, the patient was awake, alert, and oriented only to person. Her vital signs were stable with some fluctuations in blood pressure, likely secondary to her inconsistent compliance with propranolol. She was unable to follow commands or appropriately answer questions. To address any concerns regarding benzodiazepine withdrawal, which seemed to be less likely, given the absence of typical presentation (for example, tremors, restlessness, agitation, nausea, diaphoresis), the team initiated a detoxification protocol based on a symptom-triggered benzodiazepine treatment using Clinical Institute Withdrawal Assessment for Alcohol scale scoring.

The patient did not score significantly on the detox protocol. The internal medicine and neurology services were consulted. The finding of computed tomography of the patient’s head without contrast was negative, as were the results of repeat blood work and urinalysis. The patient’s repeat urine toxicology screen was unchanged from admission. The only abnormality was her ammonia level of 58 μmol/L (normal range, 11–35 μmol/L). Her electrocardiogram (ECG) showed sinus tachycardia with a QTc interval of 502 milliseconds, which was attributed to citalopram administration. After the patient’s ammonia level increased to 145 μmol/L 24 hours later, the treatment team suspected VHE, which led to discontinuation of all her medications. She was started on lactulose 20 g by mouth twice daily for 2 days, after which her ammonia level dropped to 32 μmol/L, and repeat ECG showed that her QTc interval was 454 milliseconds. Clinically, she returned to her baseline mentation. Given her reaction to VPA, she was instead started on a trial of lithium 900 mg once nightly and quetiapine 200 mg by mouth once nightly. Her lithium level after 4 days was 0.96 mmol/L, and she tolerated lithium well. By day 16, the patient was discharged with a level mood, no suicidal thoughts, and improved anxiety symptoms.

## Discussion/conclusion

One theory regarding the mechanism of action of VPA is that it blocks gamma-Aminobutyric acid (GABA) transaminase, thereby increasing GABA synthesis and release in the brain. Another theory suspects blockage of voltage-gated sodium channels and T-type calcium channels, which make VPA an anticonvulsant. VPA is a branched short-chain fatty acid that is metabolized by the liver, primarily via β-oxidation in the mitochondria. It is dependent on carnitine for entry into the mitochondria. Chronic use, high doses, and overdoses can deplete carnitine levels, leaving excess ammonia levels in the blood [2]. A smaller percentage is metabolized via omega-oxidation, which results in toxic metabolites of VPA. These metabolites can inhibit carbamoyl phosphate synthetase (CPS), which is an enzyme that normally converts ammonia to carbamoyl phosphate as part of the early steps in the urea cycle [2, 3]. Still, the question remains: What causes elevated ammonia levels in the setting of normal/therapeutic VPA levels and normal liver function? It is thought that there may be a genetic defect in CPS or ornithine transcarbamylase (OTC), which are both involved in early steps of the urea cycle. OTC deficiency is a known X-linked disorder leading to low citrulline and arginine levels along with high ammonia levels. An accumulation of ammonia is especially toxic to the central nervous system (CNS), which is unable to undergo the urea cycle. The CNS relies on conversion of ammonia to glutamine by glutamine synthetase, located in the astrocytes [2]. The accumulation of ammonia leads to increased glutamine levels that subsequently result in astrocyte swelling and cerebral edema [3]. In addition, drug–drug interactions can also play a role in elevated ammonia levels. Drugs such as phenytoin, phenobarbital, and carbamazepine can affect the activity of CPS-1 [4]. It is also theorized that drugs such as risperidone affect VPA levels by competing for binding to albumin. With coadministration, risperidone binds to albumin, leaving increased levels of VPA, effectively leading to increased ammonia levels [5]. Because the mechanism is still unclear, it remains a challenge to identify the patients who would be at greater risk in developing this adverse reaction.

Despite multiple published case reports on this subject, there is limited research into the prevalence of this phenomenon in adult patients. One study suggested that VHE is more common than previously estimated; the retrospective study of 793 patients treated with VPA showed that 2.52% of those patient showed signs and symptoms of VHE [6]. Over 20 case reports were found with the keywords “valproate,” “valproic acid,” “encephalopathy,” “ammonia,” and “hyperammonemia.” Case reports that were inaccessible, did not report VPA doses, and discussed pediatric patients or patients who recently underwent neurosurgery were excluded. A total of 12 case reports that included a sum of 17 patients were found, including 11 male and 6 female patients ranging from 20 to 61 years old. VPA was used to treat psychiatric conditions in 12 of the 17 cases, with the remainder having neurological conditions. Total daily VPA doses ranged from 500 mg to 3500 mg [4, 7–16]. Interestingly, a paper published in 2016 concluded that steady-state VPA concentrations do not appear to correlate with ammonia concentrations [17]. In fact, no relationship between dosing and severity of VHE has been found; most cases are found to have normal VPA serum levels [7]. The first set of ammonia levels drawn in the cases studied ranged from 58 to 396 μmol/L. In all the cases, the first step in treatment was discontinuation of the medication. In fact, symptoms of VHE resolved in 10 of the 17 patients just by stopping VPA. Additional therapies included hemodialysis, lactulose, l-carnitine supplementation, vitamin B, intravenous hydration, and mannitol. Of the 17 patients, 2 underwent a retrial of VPA, and both experienced recurrence of VHE symptoms [4, 7–16]. Although these treatments resulted in patients’ returning to their baseline mentation, one case resulted in a death caused by cerebral edema [8]. There are no large randomized clinical trials in regard to treatments. One literature review looked at use of levocarnitine based on the theory that carnitine depletion is the main cause of hyperammonemia, and the authors concluded that using levocarnitine can be a safe and effective treatment option, more so in the setting of acute overdose of VPA versus acute or chronic use [18].

Our patient had a positive outcome because of early detection of VHE. Her ammonia levels were drawn within 24 hours of change in her mental status, and her medications were promptly discontinued. There were no specific factors identified that could explain her susceptibility to developing encephalopathy. Once she responded within 48 hours of discontinuing the medication and initiating treatment with lactulose, it was clear that she had developed VHE.

Although it is not uncommon for patients to develop elevated ammonia levels, it can be fatal without immediate recognition and treatment for those who develop the condition. The main signs and symptoms include a sudden onset of confusion, altered mental status, and change in consciousness in the setting of normal liver function test results and elevated ammonia levels. Although there have been multiple case reports and case series published, there is a need for determining the prevalence of this condition along with standardized methods of identifying high-risk patients. The exact mechanism by which patients develop this condition is unclear, but limited research suggests it is a multifactorial process that includes genetics, drug–drug interactions, and multiple biochemical pathways. There is no established treatment protocol, but the published cases suggest early identification and immediate discontinuation of the offending agent. Clinicians prescribing VPA should be on the lookout for any sudden changes in mental status in order to provide a timely diagnosis and intervention.

## Data Availability

Background information was obtained by a literature search using PubMed. Keywords used for the search included “valproate,” “valproic acid,” “hyperammonemia,” and “hyperammonemic encephalopathy,” which led us to 18 articles that are listed in the reference list. Individual patient information was derived by Orange Regional Medical Center patient chart review.
